# Supplementary material for: Design of Hierarchical NiCo2O4 Nanocages with Excellent Electrocatalytic Dynamic for Enhanced Methanol Oxidation
Source: Nanomaterials (Basel). 2021 Oct 11;11(10):2667. doi: 10.3390/nano11102667 (PMC8539344; doi:10.3390/nano11102667)
Supplement: Supplementary file 1 [file nanomaterials-11-02667-s001.zip › nanomaterials-1389578-supplementary.pdf]

Supplementary Information

Design of Hierarchical $NiCo_2O_4$ Nanocages with Excellent Electrocatalytic Dynamic for Enhanced Methanol Oxidation

Xue Li [1,2], Gege He [3], Chong Zeng [1], Dengmei Zhou [1], Jing Xiang [1], Wenbo Chen [1], Liangliang Tian [1,*], Wenyao Yang [1,*], Zhengfu Cheng [1,*] and Jing Song [4]

1 School of Electronic Information and electrical, Chongqing University of Arts and Sciences, Chongqing 400000, China
2 School of Science, Chongqing University of Posts and Telecommunications, Chongqing 400065, China
3 School of Physics, Xi'an Jiaotong University, Shanxi 710000, China
4 Institute of Process Engineering, Chinese Academy of Sciences, Beijing 100190, China
* Correspondence: 20110061@cqwu.edu.cn (L.T.); 20160002@cqwu.edu.cn (W.Y.); 19860004@cqwu.edu.cn (Z.C.); Tel.: +86-150-2316-0415 (L.T.); +86-189-8209-6691 (W.Y.); +86-139-8384-3228 (Z.C.)

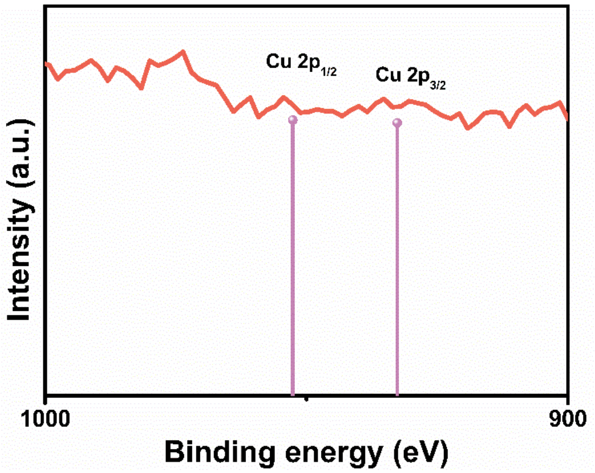

Figure S1. XPS survey of $NiCo_2O_4$.

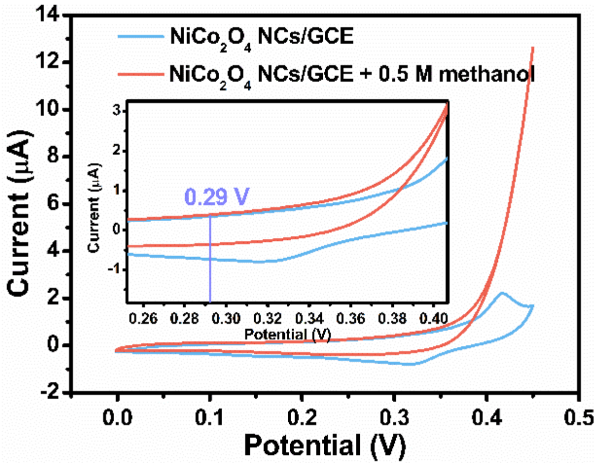

Figure S2. CV curves of $NiCo_2O_4$ NCs/GCE in 1 M KOH without methanol and with 0.5 M methanol at 50 mV s^{-1}.

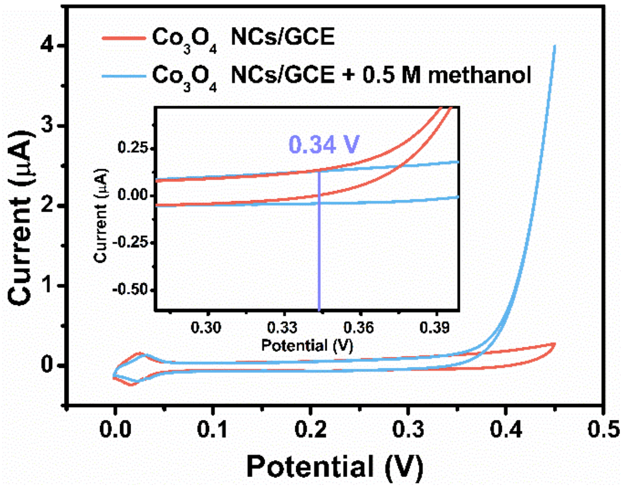

Figure S3. CV curves of Co_3O_4 NCs/GCE in 1 M KOH without methanol and with 0.5 M methanol at 50 mV s^{-1}.

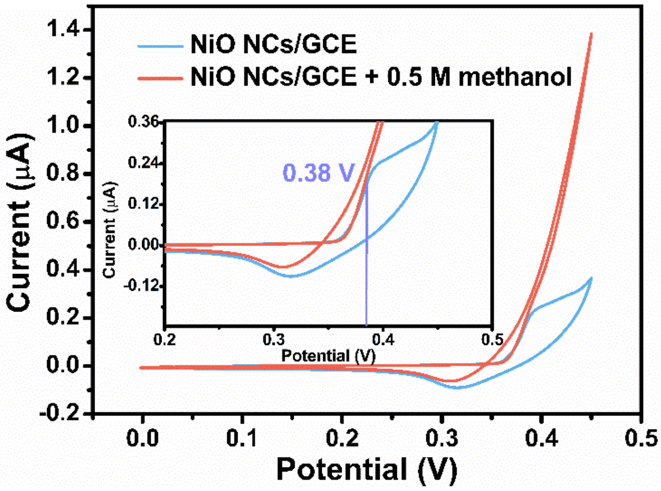

Figure S4. CV curves of NiO NCs/GCE in 1 M KOH without methanol and with 0.5 M methanol at 50 mV s^{-1}.

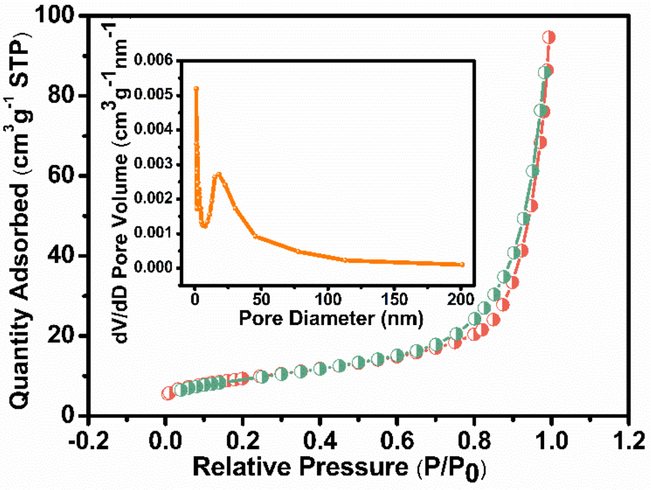

Figure S5. N_2 adsorption-desorption isotherms of the Ni-Co hydroxide precursors.
